# Blood Pressure Determinants of Cerebral White Matter Hyperintensities and Microstructural Injury: UK Biobank Cohort Study

**DOI:** 10.1161/HYPERTENSIONAHA.121.17403

**Published:** 2021-06-01

**Authors:** Karolina A. Wartolowska, Alastair J.S. Webb

**Affiliations:** Wolfson Centre for Prevention of Stroke and Dementia, Nuffield Department of Clinical Neurosciences, University of Oxford, United Kingdom.

**Keywords:** blood pressure, diffusion tensor imaging, magnetic resonance imaging, risk factors, white matter

## Abstract

Small vessel disease and related stroke and dementia risks are linked to aging and hypertension, but it is unclear whether the pulsatile or steady blood pressure (BP) component is more important for the development of macrostructural hyperintensities and microstructural white matter damage. This was a cross-sectional analysis of the UK Biobank cohort study of community-based adults from 22 UK centers. Linear associations were determined between neuroimaging markers (white matter hyperintensity [WMH] volume and diffusion imaging indices) and mean arterial pressure and pulse pressure (PP), both unadjusted and adjusted for age, sex, cardiovascular risk factors, antihypertensive medication, BP source, and assessment center. In 37 041 participants aged 45 to 82 years (53% female), univariable analyses demonstrated that increases in both BP components were associated with greater WMH volume and white matter injury on diffusion indices, with a larger effect for PP (standardized effect size for WMH: mean arterial BP: 0.182 [95% CIs, 0.170–0.193]; PP: 0.285 [95% CIs, 0.274–0.296]). In multivariable analyses, associations with mean arterial pressure remained similar, but associations with PP diminished, reflecting covariance with age and risk factors (standardized effect size for WMH: mean arterial BP: 0.106 [95% CIs, 0.095–0.117]; PP: 0.011 [95% CIs, −0.001 to 0.023]). The synergistic interaction between PP and age increased the effect of age on WMH and diffusion indices. Both macrostructural and microstructural white matter injury had similar associations with the pulsatile and steady components of hypertension, although PP accentuated the relationship between age and white matter damage.

## Abstract 2

Supplemental Digital Content is available in the text.

**See Editorial, pp 540–542**

Cerebral small vessel disease (SVD) is associated with an increased risk of stroke, vascular dementia, functional impairment, and all-cause mortality^1^ and occurs in over 50% of people over the age of 65.^2^

Direct injury to small perforating vessels cannot be readily visualized in vivo and, therefore, the extent and progress of the SVD are often estimated by assessing the damage to the brain parenchyma using neuroimaging techniques, such as magnetic resonance imaging (MRI).^1,3,4^ SVD manifests as white matter hyperintensities (WMH) on structural MRI and as microstructural changes on diffusion imaging (dMRI). Both macrostructural and microstructural damage are strongly associated with advancing age and hypertension; however, it is unclear whether the pulsatile or steady blood pressure (BP) component of hypertension is more important for the development of SVD and associated negative clinical outcomes.^5–7^ Pulse pressure (PP) is a risk factor for stroke and other cardiovascular events, independent of mean arterial pressure (MAP).^8^ Elevated PP reflects increasing arterial stiffness^9^ and transmission of pulsatile blood flow to the periphery.^10^

Raised diastolic BP (DBP) in mid-life is associated both with increased arterial stiffness^6^ and higher volume of WMH later in life.^11^ Moreover, MAP has a stronger effect on WMH volume than PP.^12^ Even the white matter outside the WMH, which appears unaffected on structural imaging, may show signs of microstructural damage.^13^ dMRI can detect subtle changes in brain structure and correlates better with clinical outcomes of SVD than other MRI measures.^14,15^ In SVD, typically, there is an increase in the magnitude of diffusion reflected by higher mean diffusivity (MD) and a decreased directionality of diffusion corresponding to lower fractional anisotropy (FA).

The aim of this study was to assess consistency in the pattern of association between, both, the pulsatile and steady-state components of BP and, both, microscopic and macroscopic white matter injury and whether these relationships may indicate a common physiological process, and which BP component may be more relevant to the development of white matter injury.

## Methods

### Participants

UK Biobank is a large cohort study including demographic, lifestyle, clinical, and imaging data from 502 540 middle-aged community-based people recruited from 22 centers across the United Kingdom.^16,17^ Eligible participants had structural brain MRI data, including both WMH volume and dMRI, as well as systolic and DBP measurements. People with a condition potentially associated with cerebral WM damage were excluded on the basis of the codes from the *International Statistical Classification of Diseases and Related Health Problems*, *Tenth Revision* (*ICD-10*), including multiple sclerosis or other demyelinating disorders, cerebral infarction, encephalitis or brain abscess, brain tumor, or systemic lupus erythematosus. All UK Biobank data used for analyses in this study are available to eligible researchers after registration and can be accessed at https://biobank.ndph.ox.ac.uk.

### Data Management

UK Biobank brain MRI data were acquired on a single Siemens Skyra 3 T scanner, and the sequence parameters have been published previously.^17^ T1-weighed 3D magnetisation-prepared 180 degrees radiofrequency pulses and rapid gradient-echo, fluid-attenuated inversion recovery, and dMRI data were preprocessed and analyzed by the UK Biobank brain imaging team using FMRIB Software Library tools (http://www.fmrib.ox.ac.uk/fsl). The analysis pipeline and quality assessment were described previously.^18^ In brief, the T1-weighted images were skull-stripped and bias field corrected using Brain Extraction Tool^19^ and segmented into white and gray matter and cerebrospinal fluid using FMRIB’s Automated Segmentation Tool.^20^

In the UK Biobank, the volume of WMH was calculated from the T1-weighted and fluid-attenuated inversion recovery images using Brain Intensity Abnormality Classification Algorithm,^21^ while the volumes of segmented white matter were estimated using FMRIB’s Automated Segmentation Tool.^20^ Both algorithms are a part of the FMRIB Software Library.^22^ The WMH load was calculated by dividing the WMH volume by the total volume of white matter and logit-transforming this ratio to normalize and stabilize the variance.

dMRI data were corrected for distortions, eddy currents, and head motion and modeled using FMRIB’s Diffusion Toolbox for diffusion modeling and tractography analysis,^23,24^ while neurite orientation dispersion and density imaging modeling was conducted using the Accelerated Microstructure Imaging via Convex Optimization tool.^25^ In the UK Biobank, tract-average diffusion tensor imaging and neurite orientation dispersion and density imaging measures are derived for 27 major white matter tracts (15 tracts, 12 of them bilateral) using AutoPtx.^26^

The diffusion tensor imaging measures included FA, corresponding to the directionality of diffusion, and MD, measuring the overall diffusivity. Diffusion tensor imaging measures are sensitive to myelination and structural integrity of white matter but cannot distinguish between loss of neurites and changes in neurite arrangement.^27^ Neurite orientation dispersion and density imaging, however, can estimate diffusion fraction corresponding to neurite density, that is, the intracellular volume fraction (ICVF) and the free water fraction, that is, isotropic compartment volume fraction (ISOVF).^25,28^ As dMRI measures in the UK Biobank are derived per white matter tract, an average value was calculated across all cerebral tracts.

The main explanatory variables were the MAP and the PP. In the UK Biobank, BP was measured twice by trained nurses after the participant had been at rest for at least 5 minutes in the seated position with a digital sphygmomanometer (Omron 705 IT; OMRON Healthcare Europe B.V., Hoofddorp, the Netherlands) with a suitably sized cuff. In this study, BP values measures were averaged within a visit. When automated BP values were missing, values from the pulse wave analysis were used.^11^ Arterial stiffness index was measured using the PulseTrace PCA2 (CareFusion, San Diego, CA) to record a 10 to 15 seconds pulse waveform by finger photoplethysmography, which were repeated on a larger finger or the thumb if less than two-thirds of the waveform were visible or if the waveform did not stabilize within 1 minute. Arterial stiffness index was derived from the interval between the forward and presumed aortic-reflected reverse-traveling pulse wave, standardized to the standing height. Patients with hypertension were identified on the basis of reported hypertension diagnosis made by a doctor. All variables and their acquisition are described on the UK Biobank showcase website (https://biobank.ndph.ox.ac.uk/showcase/).

### Statistical Analysis

To compare the explanatory variables with different units of measurement, continuous variables were centered and scaled before the regression analysis.

The associations between the concurrent values of MAP and PP and each of neuroimaging markers, including WMH, FA, MD, ICVF, and ISOVF, were analyzed using univariable, age- and sex-adjusted, and multivariable linear models adjusted for the other BP measure, age, sex, smoking status, diagnosis of diabetes, antihypertensive medication, assessment center, and the source of BP measurement. Analyses were repeated after stratification by age decade, antihypertensive medication status, WMH load quartile, and after adjusting dMRI analysis for WMH load. The multivariable models were also run with MAP and PP values from the baseline visit (adjusting for the time between the visits) and with concurrent systolic BP and DBP instead of MAP and PP. Interactions between BP and age, sex, and antihypertensive medication were also determined. Standardized coefficients between the neuroimaging measures and BP were presented in tables and forest plots. Relationships between dMRI measures and quintiles of PP and MAP were also plotted after stratifying by age decade and WMH load quartile within an age decade. Modeling assumptions were checked using diagnostic plots of residuals. Fractional polynomials terms were used to determine the presence of any statistically significant nonlinear relationships, with Bayes information criterion used to determine whether model fit had been improved or worsened through the addition or removal of any terms. Where reported, statistical significance is taken as a *P*<0.05.

### Statistical Software and Packages

All data management and analyses were performed using R version 4.0.2 using the data.table package version 1.13.6, the magrittr package version 2.0.1, and the lme4 package version 1.1.26. Figures were plotted using ggplot2 version 3.3.3 and annotated using captioner version 2.2.3. Additionally, stringr version 1.4.0 and fst 0.9.4 packages were used for data management. The article was typeset using knitr version 1.30 in RMarkdown.

## Results

Out of 38 347 UK Biobank participants with structural brain MRI data, 37 041 (96.6%) were included in the analyses; 492 (1.3%) were excluded due to confounding diagnoses and 822 (2.1%) had no BP data. The summary characteristics of the participants are presented in Table.

**Table.**
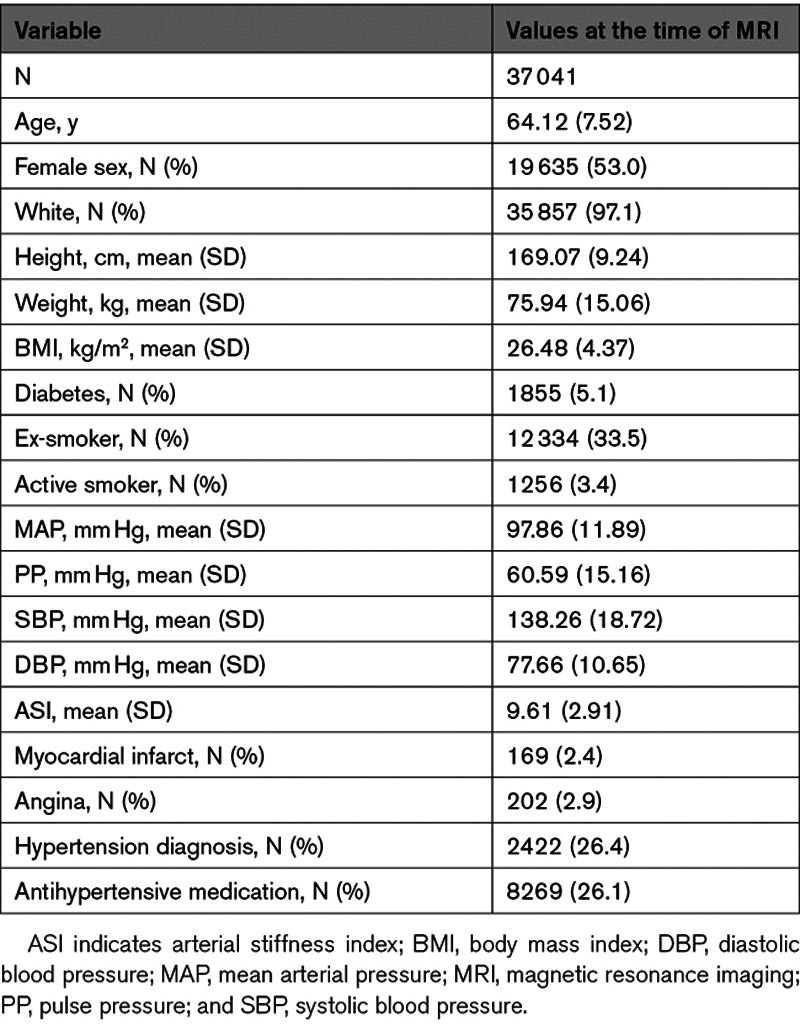
Baseline Characteristics of Participants

**Figure 1.**
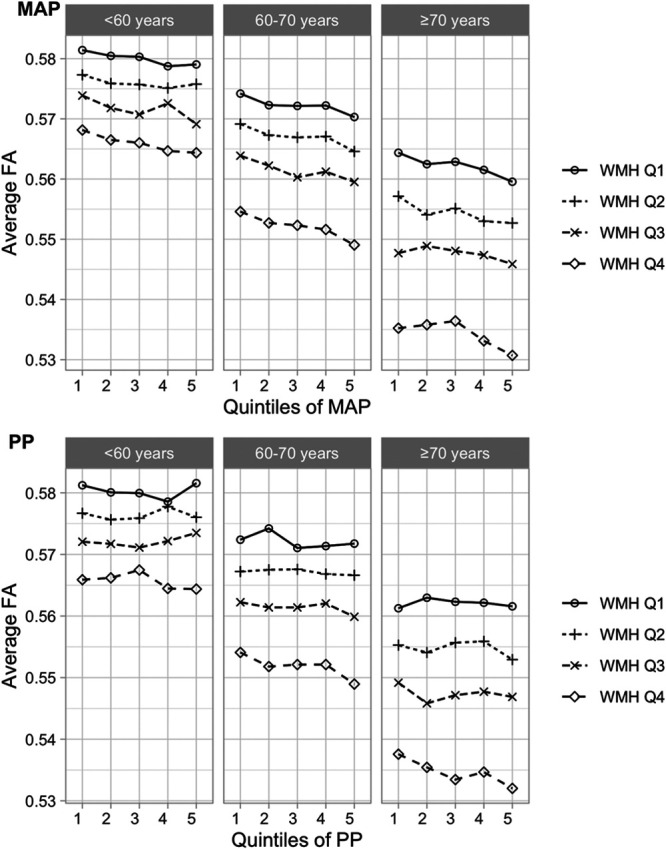
**Associations between fractional anisotropy (FA) and blood pressure.** Average FA values are plotted against quintiles of mean arterial pressure (MAP; top) and pulse pressure (PP; bottom) and stratified by quartiles of white matter hyperintensity (WMH) load and age decade.

All 4 markers of microstructural damage deteriorated with age decade and with increasing WMH load (Figure 1 and Figures S1 through S3 in the Data Supplement). PP was more strongly associated with increasing age than MAP (Figure S4).

**Figure 2.**
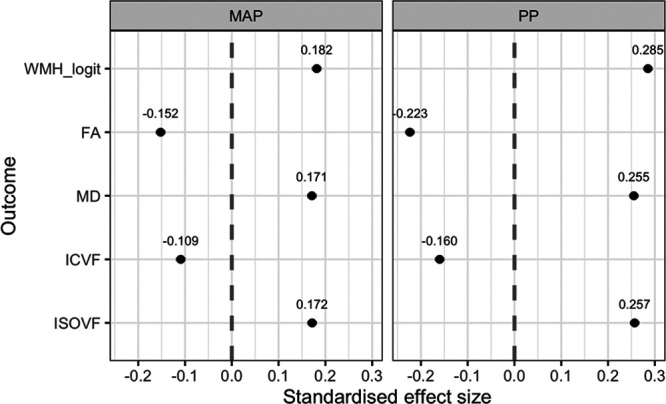
**Standardized coefficients with 95% CI for concurrent mean arterial pressure (MAP) and pulse pressure (PP) in univariable linear analyses with neuroimaging markers as outcome variables.** FA indicates fractional anisotropy; ICVF, intracellular volume fraction; ISOVF, isotropic compartment volume fraction; MD, mean diffusivity; and WMH_logit, logit-transformed white matter hyperintensity load.

In unadjusted analyses, high MAP and PP were both associated with macrostructural white matter injury reflected by WMH load and microstructural damage, including reduced directionality of diffusion measured with FA, increased MD, lower neuronal density reflected by ICVF, and increased free water diffusion measured as ISOVF (Figure 2). Standardized associations were similar when comparing macrostructural versus microstructural injury, and the pattern of associations was similar for MAP and PP, with the strongest effect size for WMH load and the weakest for ICVF. However, after adjusting for age and sex, the effect of PP on all neuroimaging indices became less marked due to the correlation between PP and age (Figure S5).

**Figure 3.**
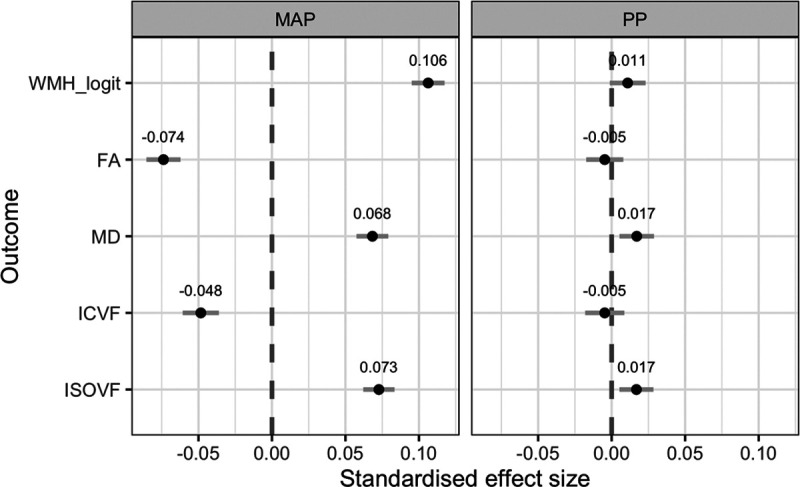
**Standardized coefficients with 95% CI for concurrent mean arterial pressure (MAP) and pulse pressure (PP) in multivariable linear analyses adjusted for the other blood pressure measure, age, sex, smoking status, diabetes, and a source of blood pressure measurement.** FA indicates fractional anisotropy; ICVF, intracellular volume fraction; ISOVF, isotropic compartment volume fraction; MD, mean diffusivity; and WMH_logit, logit-transformed white matter hyperintensity load.

In fully adjusted analyses, MAP remained associated with greater white matter injury on all neuroimaging indices, with the strongest effect on WMH load (Figure 3 and Table S1), and the pattern of associations remained similar across different age groups (Figure S6). The effect of PP remained significant only for MD and ISOVF, which reflected the overall diffusivity and free water diffusion, respectively. Age and need for antihypertensive medication were associated with worse white matter injury on macrostructural and microstructural indices and female sex had a protective effect on diffusion changes but not WMH load. As expected, all microstructural measures were more indicative of white matter injury in people with higher WMH load (Figure S7). After additionally adjusting dMRI analyses for WMH load, the effect of MAP on FA, MD, and ISOVF was greatly reduced, and its association with ICVF was no longer significant, whereas the association with ISOVF became the strongest. Adjusting for WMH load had a less marked effect on PP interactions and resulted in only a small reduction in the effect of PP on MD and ISOVF (Figures S8 and S9).

In addition to the strong direct association between age and PP, there was a significant interaction between age and PP, with PP amplifying the effect of age for all neuroimaging indices, except for ISOVF (Table S2). PP also had a synergistic effect with an increase of WMH load on all microstructural measures. Conversely, antihypertensive medication attenuated the negative effect of PP on WMH load and ISOVF (Figure S10).

Unlike PP, there were no significant interactions between the effect of MAP and age on any of the neuroimaging markers. MAP amplified the effect of WMH load on FA, MD, and ISOVF, but the standardized effect size was smaller than for PP. The interactions between MAP and antihypertensive medication were protective against worsening of FA and WMH load.

In comparison to our earlier study that demonstrated that past DBP is particularly associated with WMH later in life, historic DBP was also more strongly associated with white matter injury on dMRI indices in participants under the age of 50, but overall the association pattern of past and concurrent MAP and PP versus past and concurrent DBP and systolic BP, respectively, were similar for both WMH and dMRI indices (Figure S11).

## Discussion

### Main Findings

This large community-based study has demonstrated that both pulsatile and steady BP are associated with both microstructural white matter injury on diffusion imaging and macrostructural damage visible as WMH. The negative effect of high BP persists after correcting for the extent of macrostructural white matter lesions. After adjustment for age, cardiovascular risk factors, and antihypertensive use, MAP remained a strong predictor of microstructural and macrostructural white matter injury, but PP was weakly associated only with the overall magnitude of diffusion and free water diffusion, most likely reflecting demyelination and excess extracellular fluid. However, there was a strong synergistic interaction between PP and age, which was not evident for MAP. In contrast, the relationship between MAP and microstructural indices was more attenuated following adjustment for WMH load. Overall, the pattern of associations between BP indices and both microstructural and macrostructural markers of white matter injury was largely consistent, but PP predominantly reflected and exacerbated the relationship between age and white matter injury, whereas MAP was independent of age.

The lack of attenuation of the relationship between MAP and WMH following adjustment for age, with significant attenuation of the relationship between MAP and dMRI measures after adjustment for WMH, demonstrates the independence of MAP and age. Moreover, the pattern for MAP was consistent between WMH load and dMRI measures, which is in line with the hypothesis that mechanisms underlying macrostructural and microstructural white matter changes are similar. FA, MD, and WMH share a genetic component which suggests that there is a shared polygenic component involved in the mechanisms underlying white matter damage in SVD.^29^ It is also believed that microstructural changes precede WMH development,^30^ but the cross-sectional nature of this study precluded any analysis of the temporal relationship between the microstructural and macrostructural changes. However, these results suggest that dMRI measures may be a more sensitive marker of white matter injury and may enable us to investigate the underlying processes in greater detail than is revealed by established WMH.

PP covaried with age so adjustment for age and cardiovascular risk factors attenuated the relationships with WMH load and diffusion indices. There was also a strong interaction between PP and age, which implies that although PP is a marker of increasing vascular age, it also reinforces the effect of age and may represent one mechanism by which WMH load increases with age. As dMRI indices become worse with increasing WMH load, and PP and MAP amplify this effect, PP may still play a significant role in the causation of WMH and dMRI changes at older ages. It is important to note that the magnitude of the synergistic effect was larger for PP, which suggests an involvement of PP in white matter damage, which is consistent with the literature emphasizing the role of PP as a risk factor for cardiovascular events and stroke.^6,8^

Some of the microstructural changes were related to the diffusion changes within the macrostructural lesions. This was particularly marked for the effect of MAP on ICVF, which disappeared after adjusting the analysis for WMH load. The pattern of associations with other markers of microstructural damage did not change but the strength of the associations diminished, especially for MAP. Adjusting for WMH load only minimally reduced the effect size of the PP on MD and ISOVF and the effect of PP on these 2 measures was present even in people with minimal WMH load. This suggests that microstructural changes resulting in increased overall diffusivity (MD), and especially the free water fraction (ISOVF), may be present before the development of macrostructural changes. This is in line with other studies that demonstrated that MD was an earlier marker of white matter damage that was increased even in people with very few WMH lesion and was superior to FA at differentiating between WMH and normal-appearing white matter.^31^

MAP was associated with worsening of all neuroimaging indices, except for the ICVF, whereas PP was related only to an increase in overall diffusivity and the extracellular water fraction. This suggests that an increase in MAP and PP affects the free water volume fraction rather than the intracellular fraction and that the mechanisms involved in an increase in MD and ISOVF differ from those responsible for higher WMH load and loss of directionality of diffusion (FA). It is possible that PP is responsible for demyelination and increased free water^13^ while MAP is associated with changes to the free water fraction as well as neurite organization, which would explain the association between MAP and WMH and FA. An increase in MD with increasing BP values, without the concurrent reduction in FA, may reflect higher BP causing increased extracellular fluid before actual cytoarchitectural damage.^32^ This suggests that in early SVD the organization of neurites is preserved, more than suggested by conventional FA, and that the diffusivity changes reflect free water changes, most likely demyelination,^13^ which is consistent with our findings.

Although people who required antihypertensive medication had generally higher WMH load and worse dMRI measures, in line with earlier studies,^33^ antihypertensive medication protected against a negative effect of MAP on FA and WMH load and effect of PP on ISOVF and WMH load. This is consistent with the results of trials of BP-lowering medications and their effect on WMH load^34^ but may reflect different effects of the medication on PP and MAP.^35^

### Strengths and Limitations

The main strength of this study is the large sample size of population-based participants and a fully automated analysis of the MRI data. However, there were also some limitations. First, dMRI values were averaged across all regions without any adjustments, such as weighting for the size of the region. However, effects were highly consistent between regions within the anterior circulation. Second, dMRI data were not masked for WMH, therefore, reflecting dMRI values both in the WMH and normal-appearing white matter. However, the pattern of associations between PP and MAP with dMRI measures in patients with no WMH was similar. Third, brachial PP may not be a good surrogate for intracranial arterial pulsatility.^36^ Moreover, the WMH measurement included both deep and periventricular WMH, which may not be affected by the SVD pathology in the same way. Future releases of UK Biobank data with further Brain Intensity Abnormality Classification Algorithm analysis will enable us to determine the different effects on periventricular versus deep white matter. Although we excluded patients with diagnosed conditions, which were likely to be associated with WMH, it is possible that not all WMH were due to cardiovascular causes. Similarly, a causative role of BP components would be better supported by longitudinal MRI scans, but this has so far only been released for a small fraction of participants. It is important to note that the analyses were based on linear models, although some relationships were nonlinear. However, nonlinear models did not explain a markedly greater proportion of the variance in neuroimaging markers. Finally, this study focused on structural damage, but functional changes, especially failure of small vessels’ endothelium, play an important role in development of white matter damage^1^ and hypertension associated with premature vascular aging.^37^

In conclusions, both MAP and PP are significantly associated with microstructural and macrostructural white matter injury, suggesting a common pathophysiological process for these manifestations of SVD and supporting the utility of dMRI as a sensitive marker of white matter injury. Both the pulsatile and steady BP affected the magnitude of diffusivity most likely through an increase of the extracellular water; while the steady BP was associated with the damage to neurite organization and development of white matter lesions. However, MAP was largely independent of age, whereas PP covaried with age and acted synergistically to exacerbate the age-associated worsening of SVD. This is consistent with the hypothesis that sustained hypertension contributes to white matter injury at all ages, developing into highly pulsatile BP in late life that is increasingly relevant at older ages.

### Perspectives

This is a large study of associations between BP and white matter damage in 37 041 middle-aged, community-based people; therefore, sufficiently powered to address the relative roles of steady versus pulsatile BP across age ranges. It is the first study to compare consequences of BP on both macrostructural and microstructural changes, particularly using both standard diffusion measures but also neurite orientation dispersion and density imaging, which allows to differentiate between axonal damage and changes in extracellular water.

This study demonstrated that both pulsatile and steady components of BP were associated with white matter injury on diffusion imaging as higher MD and the diffusivity fraction corresponding to extracellular water, while the high steady BP was predominantly linked to changes in neurite organization and higher WMH volume.

Further studies are required to investigate the longitudinal associations between the BP and white matter damage and how they are affected by individual antihypertensive drug classes, and whether they are linked with future clinical events.

## Acknowledgments

K.A. Wartolowska, MD, DPhil reports Clinical Research Fellow, Wolfson Centre for Prevention of Stroke and Dementia, Nuffield Department of Clinical Neurosciences, University of Oxford; AJSW, BMBCh, MRCP, DPhil): Wellcome Trust Clinical Research Career Development Fellow/Honorary Neurology Consultant, Wolfson Centre for Prevention of Stroke and Dementia, Nuffield Department of Clinical Neurosciences, University of Oxford. K.A. Wartolowska designed and performed the analysis of the data and wrote the article. K.A. Wartolowska had full access to all the data in the study and takes responsibility for the integrity of the data and the accuracy of the data analysis. A.J.S. Webb initiated, designed, and supervised the study and revised the article for important intellectual content. Both authors read and approved the final version of the article.

## Sources of Funding

This work was supported by an Alzheimer's Society grant (450-AS-PG-18-018) and by the Wellcome Trust (Wellcome Trust Clinical Research Career Development Fellowship, 206589/Z/17/Z). For the purpose of open access, a CC BY licence is applied to Author Accepted Manuscript arising from this submission, in accordance with the grant's open access conditions. The funders of the study had no role in the study design and of the study; collection, management, analysis, and interpretation of the data; preparation, review or approval of the manuscript; and decision to submit the manuscript for publication.

## Disclosures

None.

## Supplementary Material



## Novelty and Significance

### What Is New?

- This is the first study to compare the consequences of steady versus pulsatile blood pressure on macrostructural and microstructural white matter changes using both standard diffusion measures and neurite orientation dispersion and density imaging.
- Both blood pressure components increased mean diffusivity and the diffusivity fraction corresponding to extracellular water.
- High steady blood pressure was also associated with damage to neurite organization and higher white matter hyperintensity volume.
- Pulse pressure primarily accentuated the relationship between age and white matter damage.

### What Is Relevant?

- Control of both components of blood pressure may be important to prevent development of white matter injury and its deterioration with age.

### Summary

Pulsatile and steady blood pressure components were associated with white matter damage, with pulse pressure augmenting the effect of age.
