# Supplementary material for: Blood Pressure Determinants of Cerebral White Matter Hyperintensities and Microstructural Injury: UK Biobank Cohort Study
Source: Hypertension. 2021 Jun 1;78(2):532–9. doi: 10.1161/HYPERTENSIONAHA.121.17403 (PMC8260341; doi:10.1161/HYPERTENSIONAHA.121.17403)
Supplement: Supplementary file 1 [file hyp-78-532-s001.pdf]

DATA SUPPLEMENT

Blood pressure determinants of cerebral white matter hyperintensities and microstructural injury: UK Biobank cohort study.

Karolina A. Wartolowska and Alastair J.S. Webb

Affiliations: Wolfson Centre for Prevention of Stroke and Dementia, Nuffield Department of Clinical Neurosciences, University of Oxford, UK

Address: Wolfson Centre for Prevention of Stroke and Dementia, Wolfson Building, John Radcliffe Hospital, Headley Way, Oxford, OX3 9DU, UK

Key Words: small vessel disease; mean arterial blood pressure; pulse pressure; hypertension; white matter hyperintensities; diffusion tensor imaging (DTI); neurite orientation dispersion and density imaging (NODDI)

Correspondence to:

Dr Karolina Wartolowska

Wolfson Centre for Prevention of Stroke and Dementia

Nuffield Department of Clinical Neurosciences, University of Oxford

John Radcliffe Hospital, Headley Way, Oxford, OX3 9DU, UK

Tel: +44 1865 234 340

Fax: +44 1865 234 629

Email: karolina.wartolowska@ndcn.ox.ac.uk

Table S1: **Effect sizes in the in multivariable analyses without the interactions.** The values represent standardised coefficients and their 95% confidence intervals.

Variable	FA	MD	ICVF	ISOVF	WMH
MAP	-0.033 (-0.044 to -0.023)	0.027 (0.017 to 0.037)	-0.008 (-0.019 to 0.004)	0.046 (0.036 to 0.057)	0.106 (0.095 to 0.117)
PP	-0.001 (-0.013 to 0.011)	0.013 (0.002 to 0.024)	-0.001 (-0.013 to 0.012)	0.014 (0.003 to 0.026)	0.010 (-0.002 to 0.022)
Age	-0.213 (-0.225 to -0.201)	0.298 (0.288 to 0.309)	-0.122 (-0.134 to -0.109)	0.367 (0.356 to 0.378)	0.475 (0.464 to 0.485)
Sex	0.041 (0.022 to 0.059)	-0.248 (-0.265 to -0.231)	0.132 (0.112 to 0.152)	-0.199 (-0.217 to -0.182)	0.078 (0.059 to 0.097)
WMH	-0.384 (-0.394 to -0.373)	0.391 (0.381 to 0.401)	-0.386 (-0.398 to -0.375)	0.251 (0.240 to 0.261)	.
Antihypertensives	-0.140 (-0.163 to -0.117)	0.119 (0.098 to 0.139)	-0.026 (-0.050 to -0.002)	0.183 (0.162 to 0.205)	0.236 (0.213 to 0.259)

Table S2: **Interactions between the effect of age and blood pressure on dMRI measures in fully-adjusted analyses.** The values represent standardised coefficients and their 95% confidence intervals.

Variable	FA	MD	ICVF	ISOVF	WMH
MAP	-0.042 (-0.060 to -0.025)	0.041 (0.025 to 0.057)	-0.012 (-0.031 to 0.006)	0.064 (0.047 to 0.081)	0.116 (0.098 to 0.134)
PP	-0.001 (-0.020 to 0.018)	0.019 (0.002 to 0.036)	0.001 (-0.019 to 0.021)	0.022 (0.004 to 0.041)	0.006 (-0.014 to 0.026)
Age	-0.219 (-0.230 to -0.207)	0.306 (0.296 to 0.317)	-0.129 (-0.142 to -0.117)	0.371 (0.360 to 0.383)	0.474 (0.463 to 0.485)
Age*MAP	-0.003 (-0.015 to 0.009)	0.010 (-0.001 to 0.021)	-0.009 (-0.021 to 0.004)	0.005 (-0.006 to 0.016)	-0.003 (-0.014 to 0.007)
Age*PP	-0.015 (-0.027 to -0.002)	0.018 (0.007 to 0.029)	-0.019 (-0.032 to -0.006)	0.006 (-0.006 to 0.017)	0.011 (0.000 to 0.022)
Female Sex	0.047 (0.028 to 0.065)	-0.256 (-0.273 to -0.239)	0.140 (0.120 to 0.160)	-0.204 (-0.222 to -0.186)	0.080 (0.060 to 0.099)
Female Sex*MAP	-0.007 (-0.028 to 0.013)	-0.009 (-0.028 to 0.010)	-0.013 (-0.035 to 0.009)	-0.025 (-0.044 to -0.005)	-0.008 (-0.029 to 0.014)
Female Sex*PP	0.023 (0.002 to 0.044)	-0.034 (-0.053 to -0.015)	0.034 (0.011 to 0.056)	-0.014 (-0.034 to 0.005)	0.025 (0.004 to 0.047)
WMH	-0.382 (-0.393 to -0.371)	0.389 (0.379 to 0.399)	-0.385 (-0.397 to -0.374)	0.249 (0.239 to 0.259)	.
WMH*MAP	-0.019 (-0.031 to -0.008)	0.017 (0.006 to 0.027)	-0.011 (-0.024 to 0.001)	0.015 (0.003 to 0.026)	.
WMH*PP	-0.051 (-0.063 to -0.039)	0.069 (0.058 to 0.080)	-0.060 (-0.073 to -0.047)	0.049 (0.037 to 0.060)	.
Antihypertensives	-0.148 (-0.172 to -0.125)	0.124 (0.103 to 0.146)	-0.027 (-0.052 to -0.002)	0.192 (0.170 to 0.215)	0.248 (0.224 to 0.273)
Antihypertensives*MAP	0.039 (0.015 to 0.064)	-0.020 (-0.042 to 0.003)	0.027 (0.000 to 0.053)	-0.012 (-0.036 to 0.012)	-0.029 (-0.055 to -0.004)
Antihypertensives*PP	0.001 (-0.022 to 0.024)	-0.012 (-0.033 to 0.009)	-0.014 (-0.039 to 0.010)	-0.031 (-0.053 to -0.009)	-0.038 (-0.062 to -0.014)

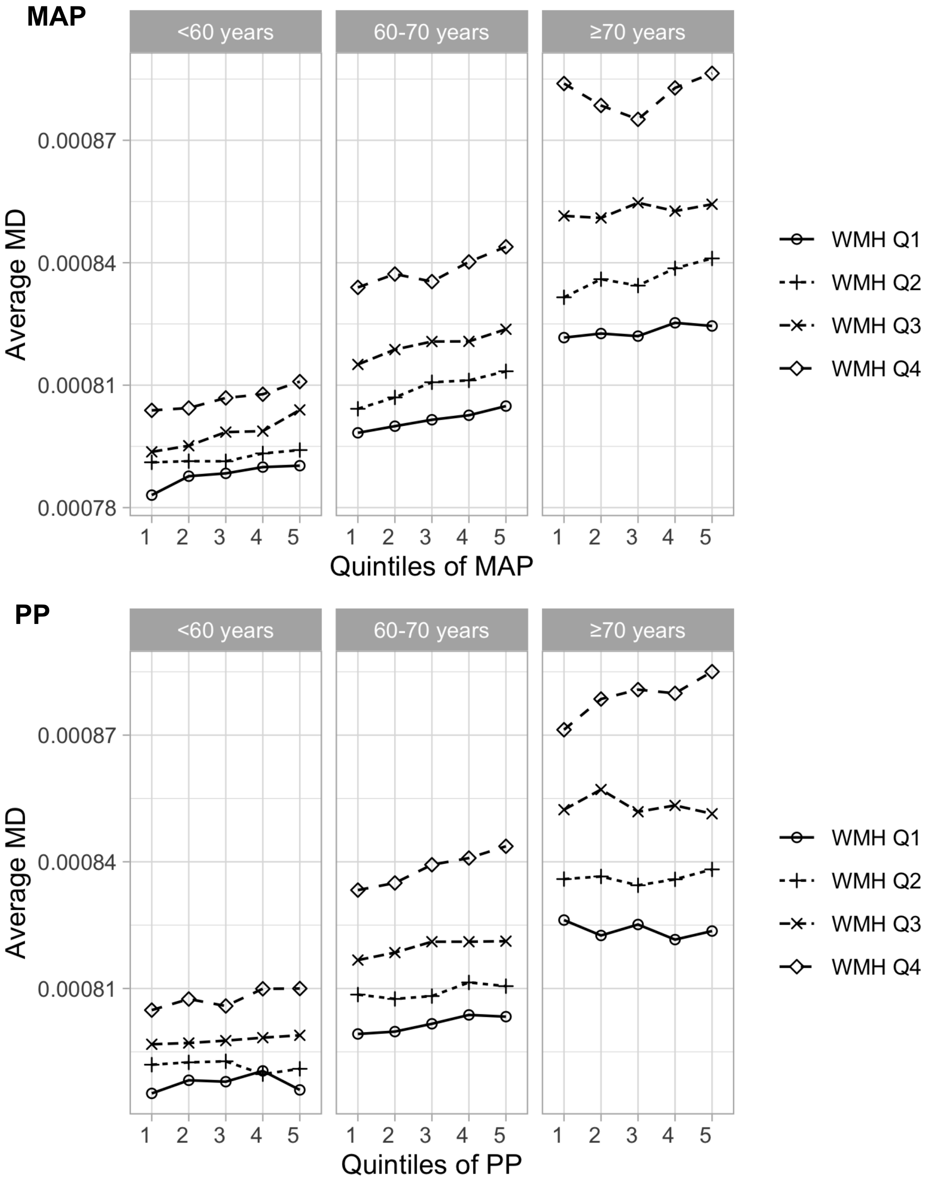

Figure S1: Mean Diffusivity (MD) plotted against quintiles of MAP (top figure) and PP (bottom figure) and stratified by quartiles of WMH load and age decade. MD - Mean Diffusivity, MAP - Mean Arterial Pressure, PP - Pulse Pressure, WMH - white matter hyperintensity load.

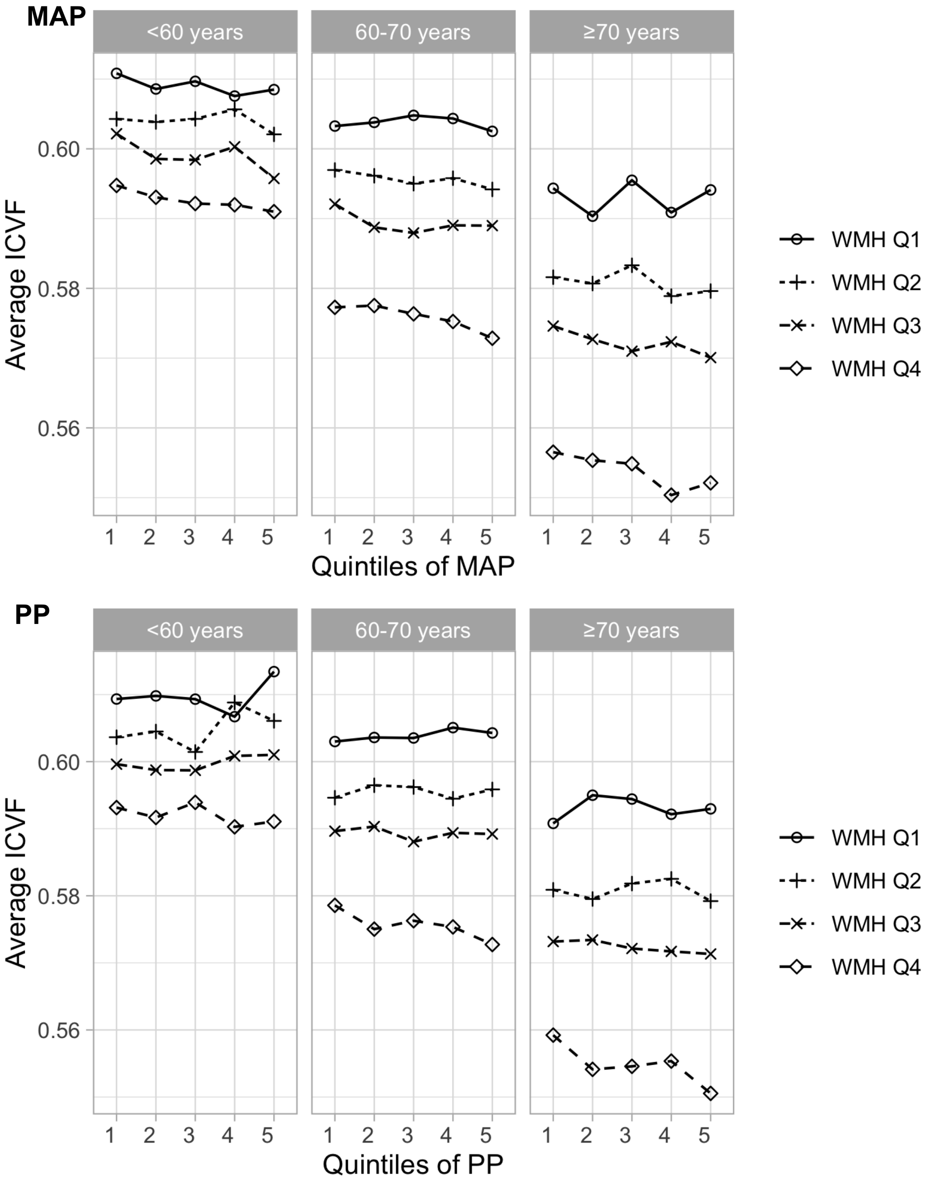

Figure S2: Intracellular Volume Fraction (ICVF) plotted against quintiles of MAP (top figure) and PP (bottom figure) and stratified by quartiles of WMH load and age decade. MAP - Mean Arterial Pressure, PP - Pulse Pressure, WMH - white matter hyperintensity load.

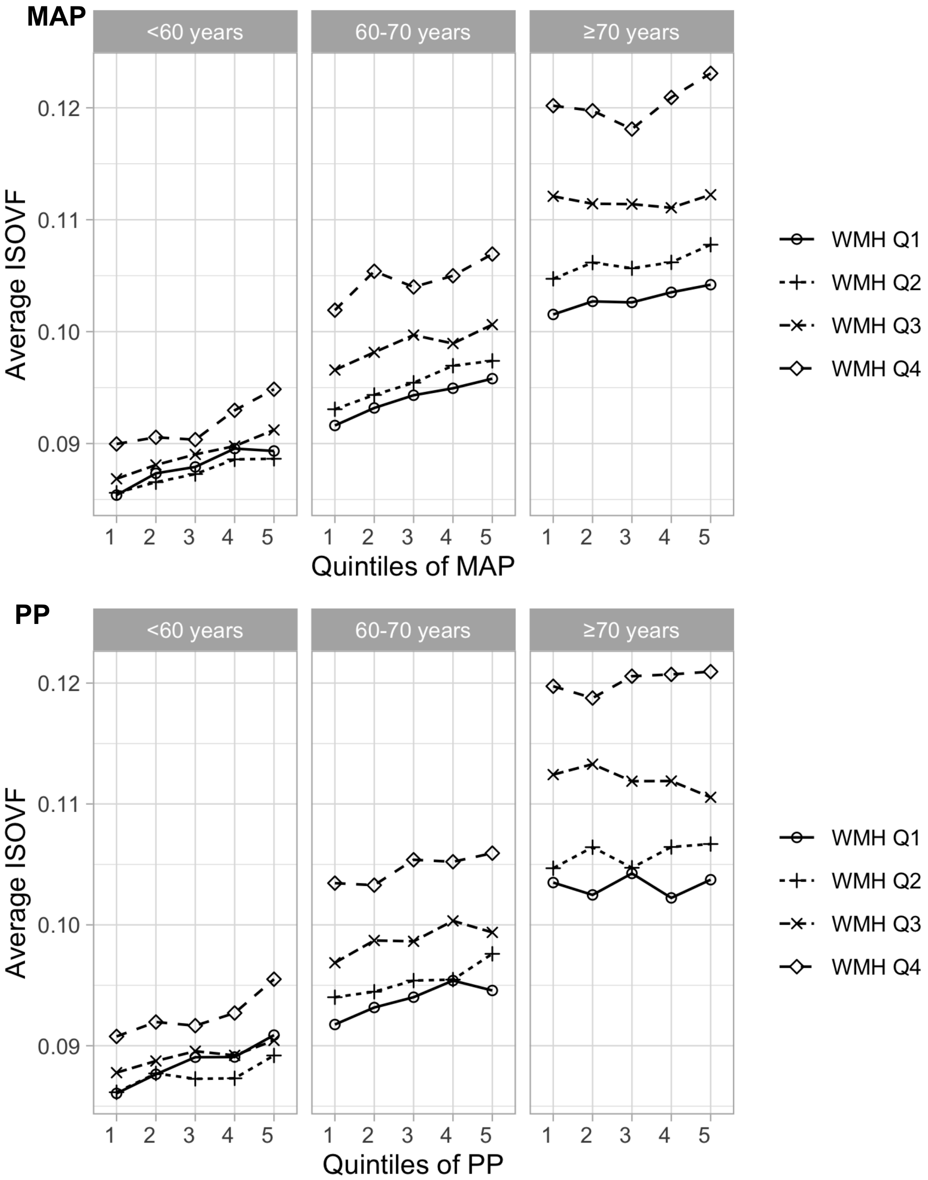

Figure S3: Isotropic Compartment Volume Fraction (ISOVF) plotted against quintiles of MAP (top figure) and PP (bottom figure) and stratified by quartiles of WMH load and age decade. MAP - Mean Arterial Pressure, PP - Pulse Pressure, WMH - white matter hyperintensity load.

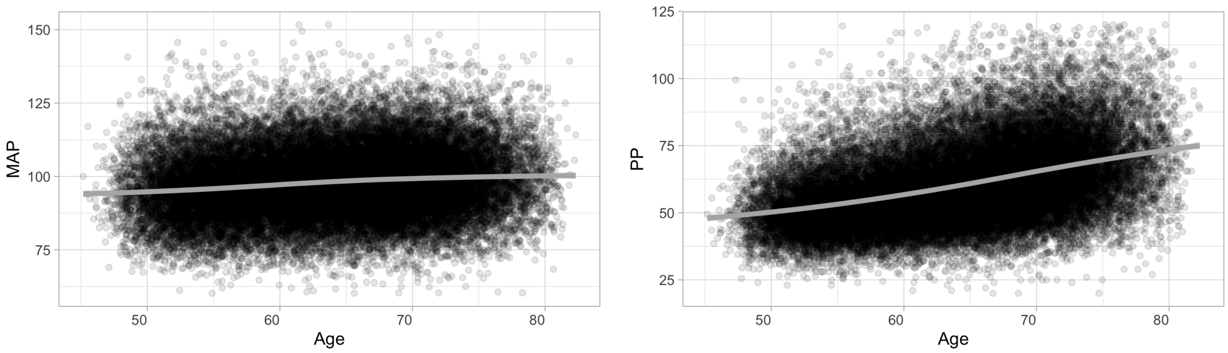

Figure S4: Mean arterial pressure (MAP) and pulse pressure (PP) distribution with age. The blue line represents a LOESS fit.

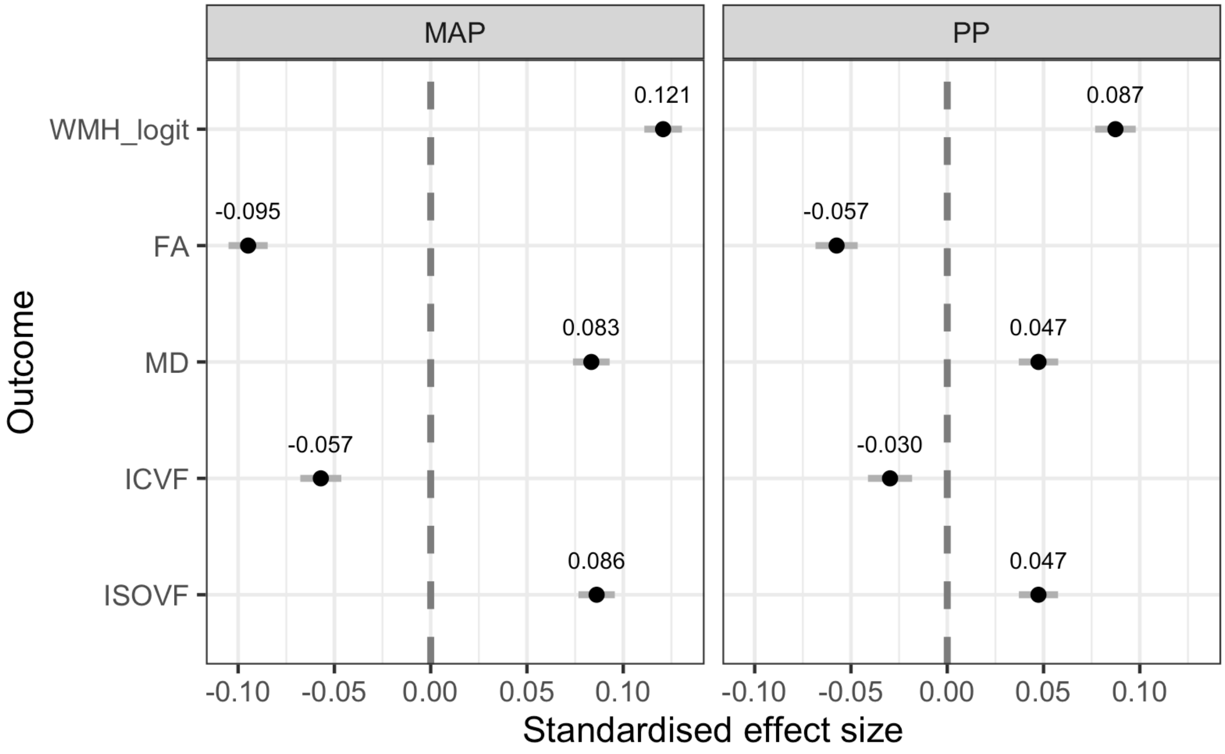

Figure S5: Standardised coefficients for concurrent mean arterial pressure (MAP) and pulse pressure (PP) in age- and sex-adjusted analyses with neuroimaging markers as outcome variables. Abbreviations: WMH_logit - logit-transformed white matter hyperintensity load, FA - Fractional Anisotropy, MD - Mean Diffusivity, ICVF - Intracellular Volume Fraction, ISOVF - Isotropic Compartment Volume Fraction.

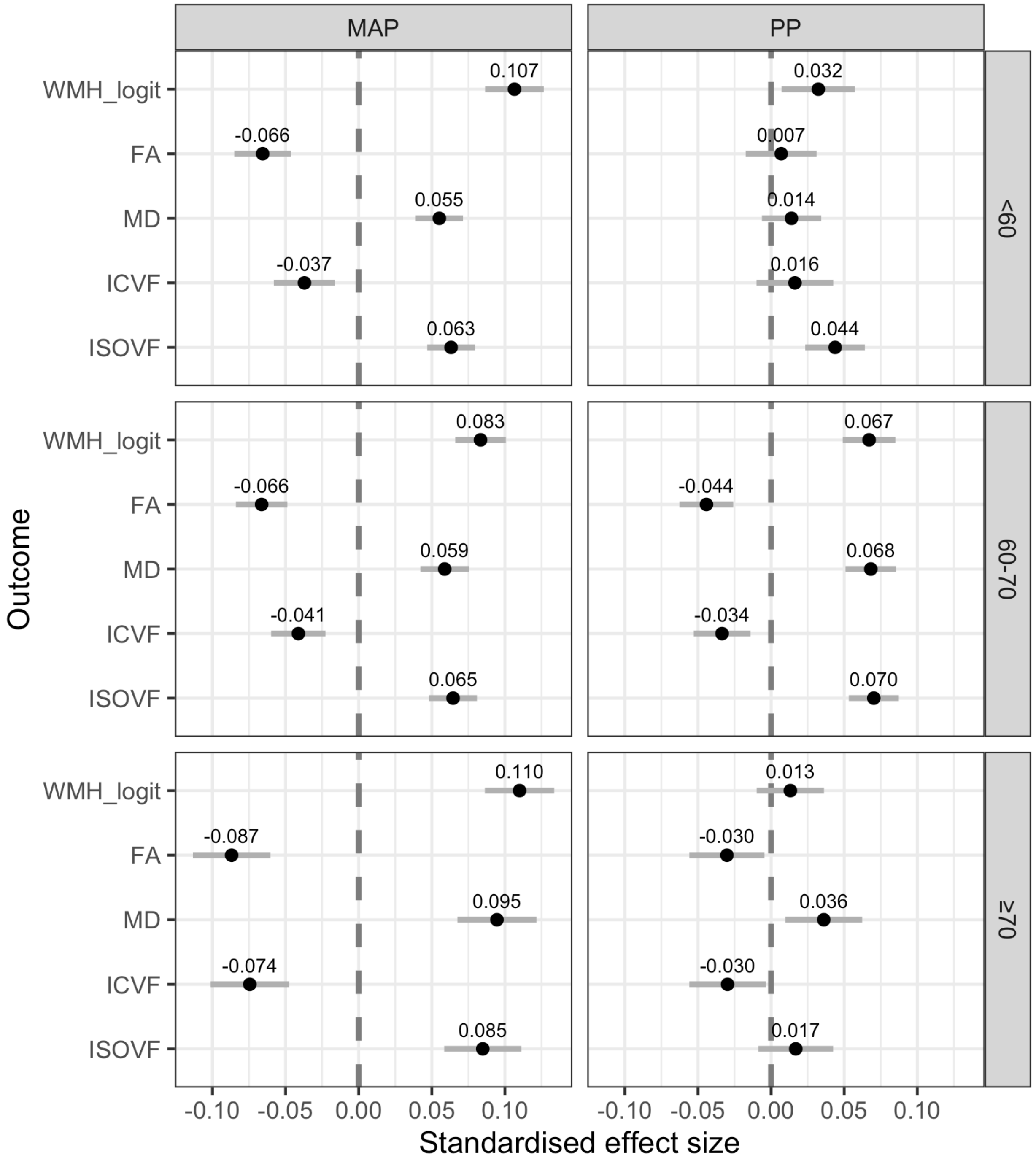

Figure S6: Standardised coefficients for concurrent mean arterial pressure (MAP) and pulse pressure (PP) in multivariable analyses adjusted for the the other blood pressure measure, sex, smoking status, diabetes, source of blood pressure measurement and stratified by age decade and not adjusted for WMH load). Abbreviations: WMH_logit - logit-transformed white matter hyperintensity load, FA - Fractional Anisotropy, MD - Mean Diffusivity, ICVF - Intracellular Volume Fraction, ISOVF - Isotropic Compartment Volume Fraction.

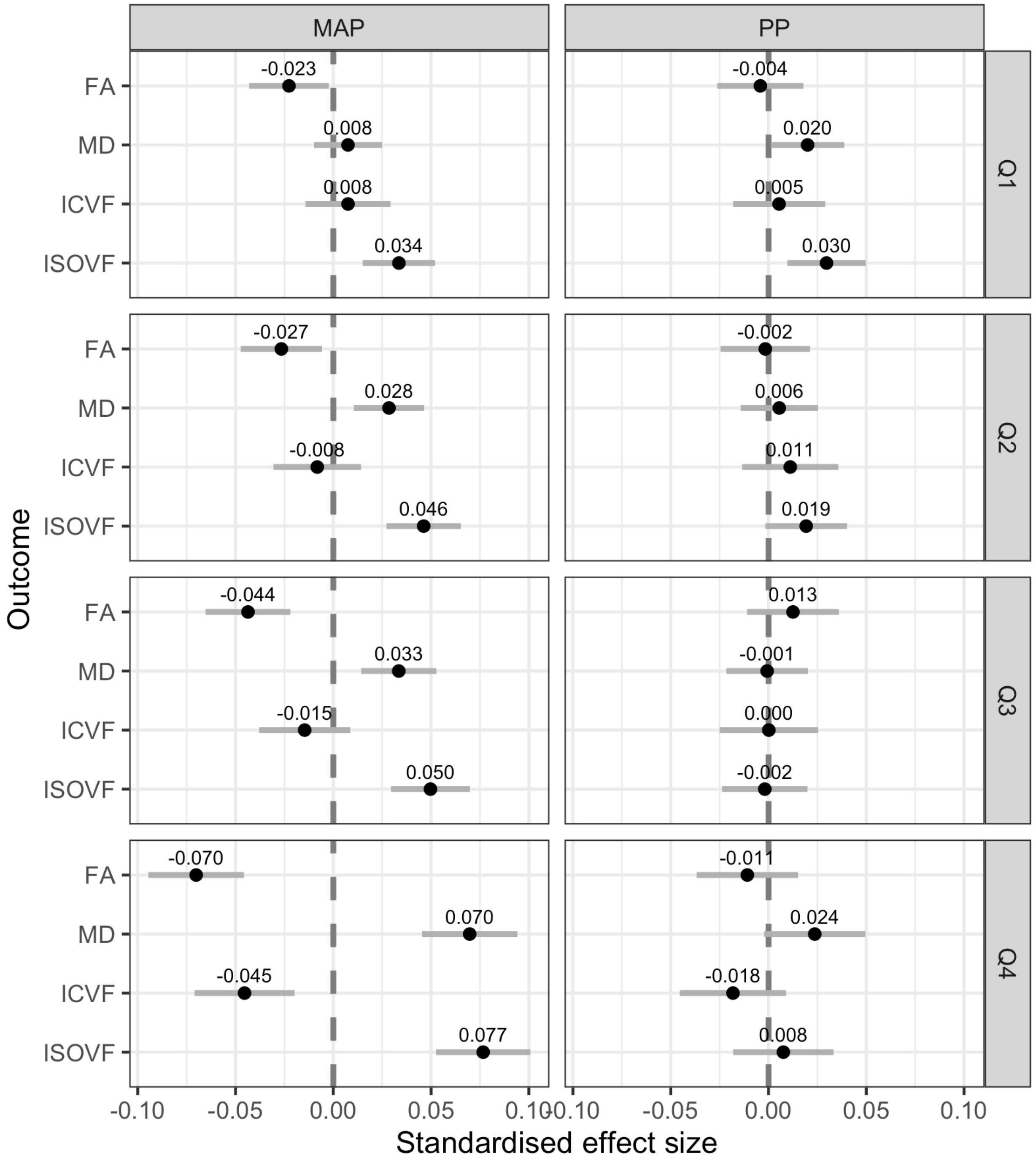

Figure S7: Standardised coefficients for concurrent mean arterial pressure (MAP) and pulse pressure (PP) in multivariable analyses adjusted for the the other blood pressure measure, age, sex, smoking status, diabetes, source of blood pressure measurement and stratified by the WMH load quartile. Abbreviations: WMH - white matter hyperintensity load, FA - Fractional Anisotropy, MD - Mean Diffusivity, ICVF - Intracellular Volume Fraction, ISOVF - Isotropic Compartment Volume Fraction.

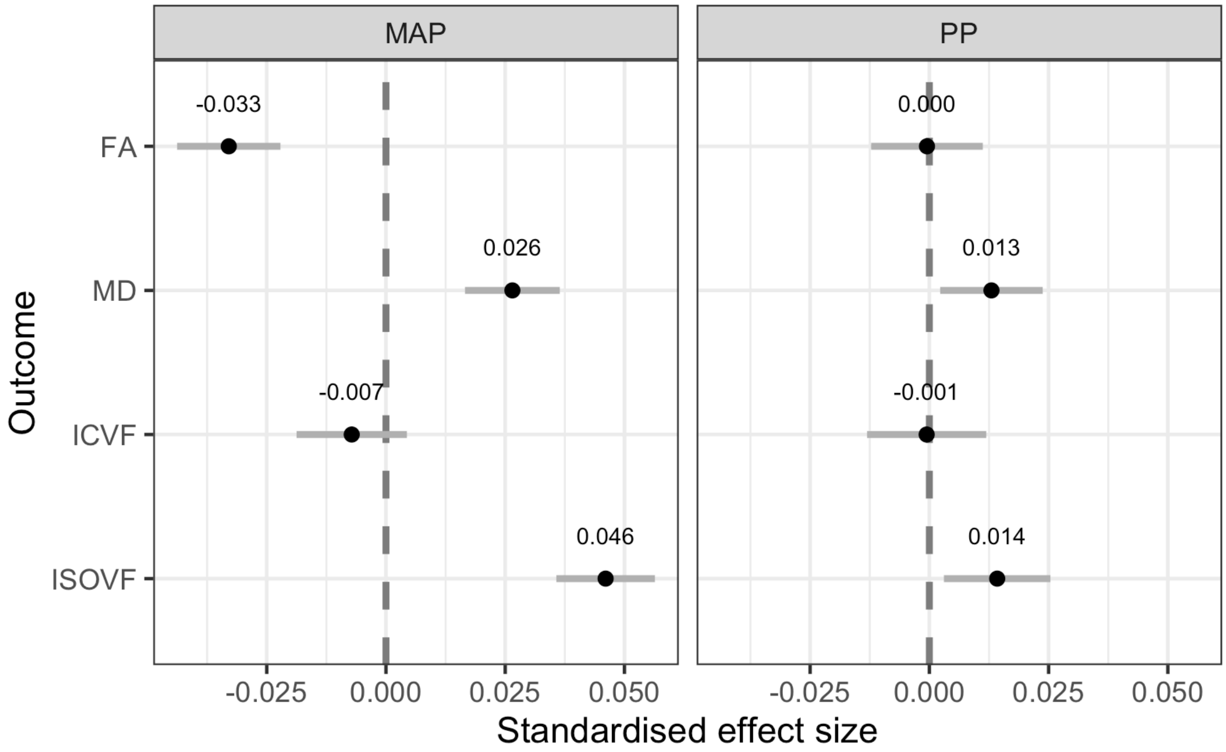

Figure S8: Standardised coefficients for concurrent mean arterial pressure (MAP) and pulse pressure (PP) in multivariable analyses adjusted for the WMH load, the other blood pressure measure, age, sex, smoking status, diabetes, source of blood pressure measurement. Abbreviations: WMH_logit - logit-transformed white matter hyperintensity load, FA - Fractional Anisotropy, MD - Mean Diffusivity, ICVF - Intracellular Volume Fraction, ISOVF - Isotropic Compartment Volume Fraction.

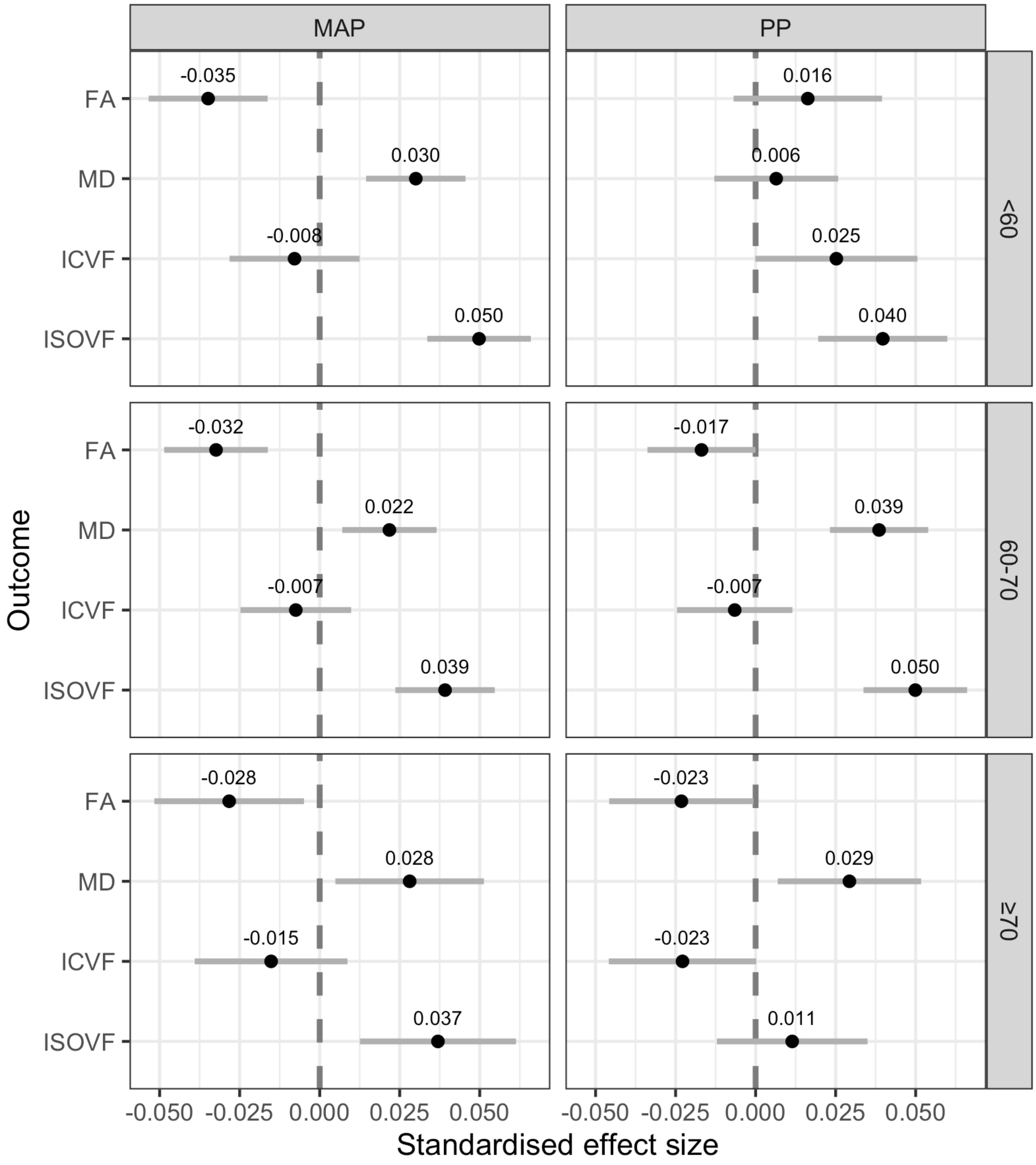

Figure S9: Standardised coefficients for concurrent mean arterial pressure (MAP) and pulse pressure (PP) in multivariable analyses adjusted for the WMH load, the other blood pressure measure, sex, smoking status, diabetes, source of blood pressure measurement, and assessment centre and stratified by age decade. Abbreviations: WMH_logit - logit-transformed white matter hyperintensity load, FA - Fractional Anisotropy, MD - Mean Diffusivity, ICVF - Intracellular Volume Fraction, ISOVF - Isotropic Compartment Volume Fraction.

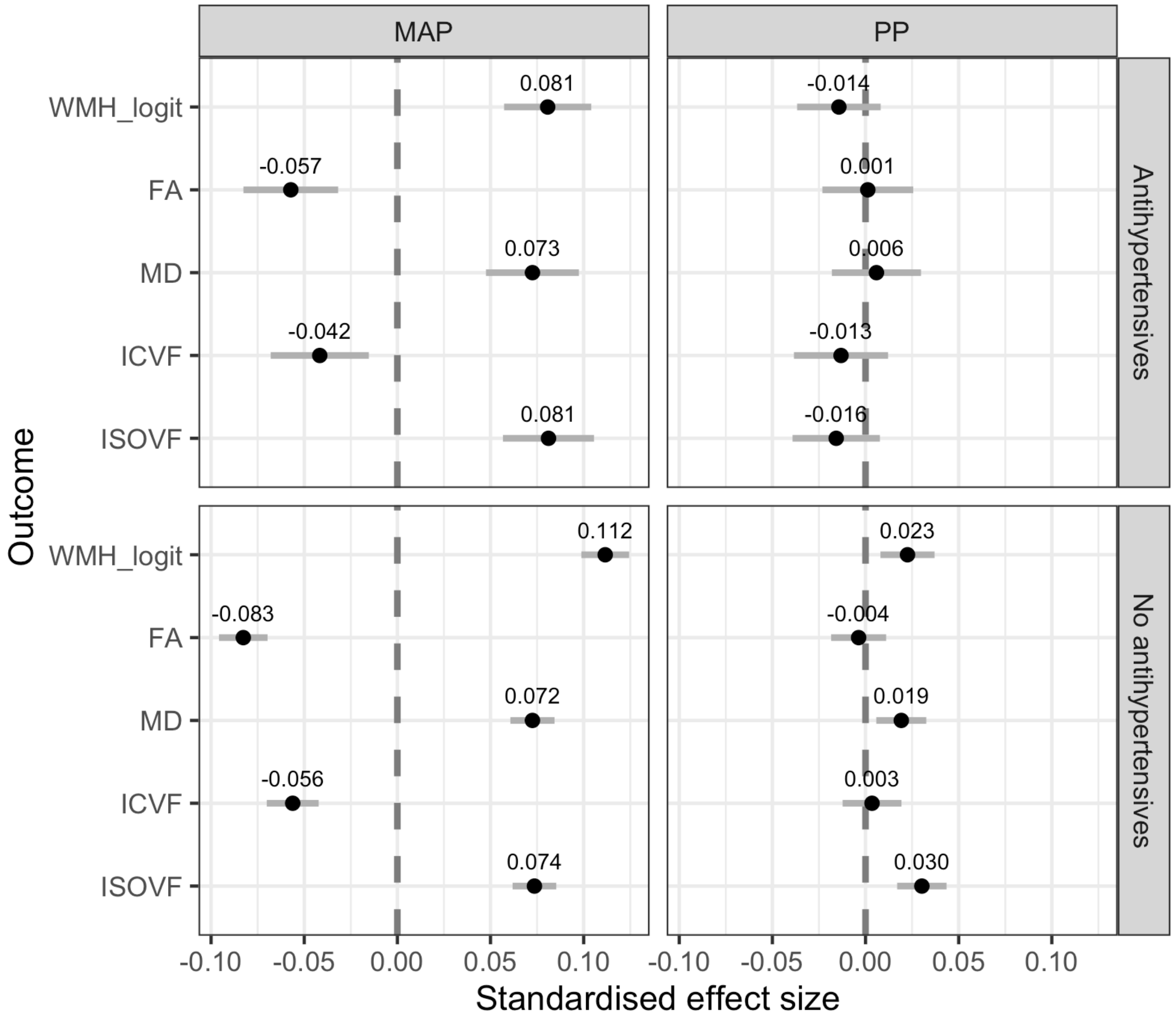

Figure S10: Standardised coefficients for mean arterial blood pressure (MAP) and pulse pressure (PP) in multivariable analyses adjusted for the other blood pressure measure, age, sex, smoking status, diabetes, source of blood pressure measurement, and assessment centre and stratified by antihypertensive medication status. Abbreviations: WMH_logit - logit-transformed white matter hyperintensity load, FA - Fractional Anisotropy, MD - Mean Diffusivity, ICVF - Intracellular Volume Fraction, ISOVF - Isotropic Compartment Volume Fraction.

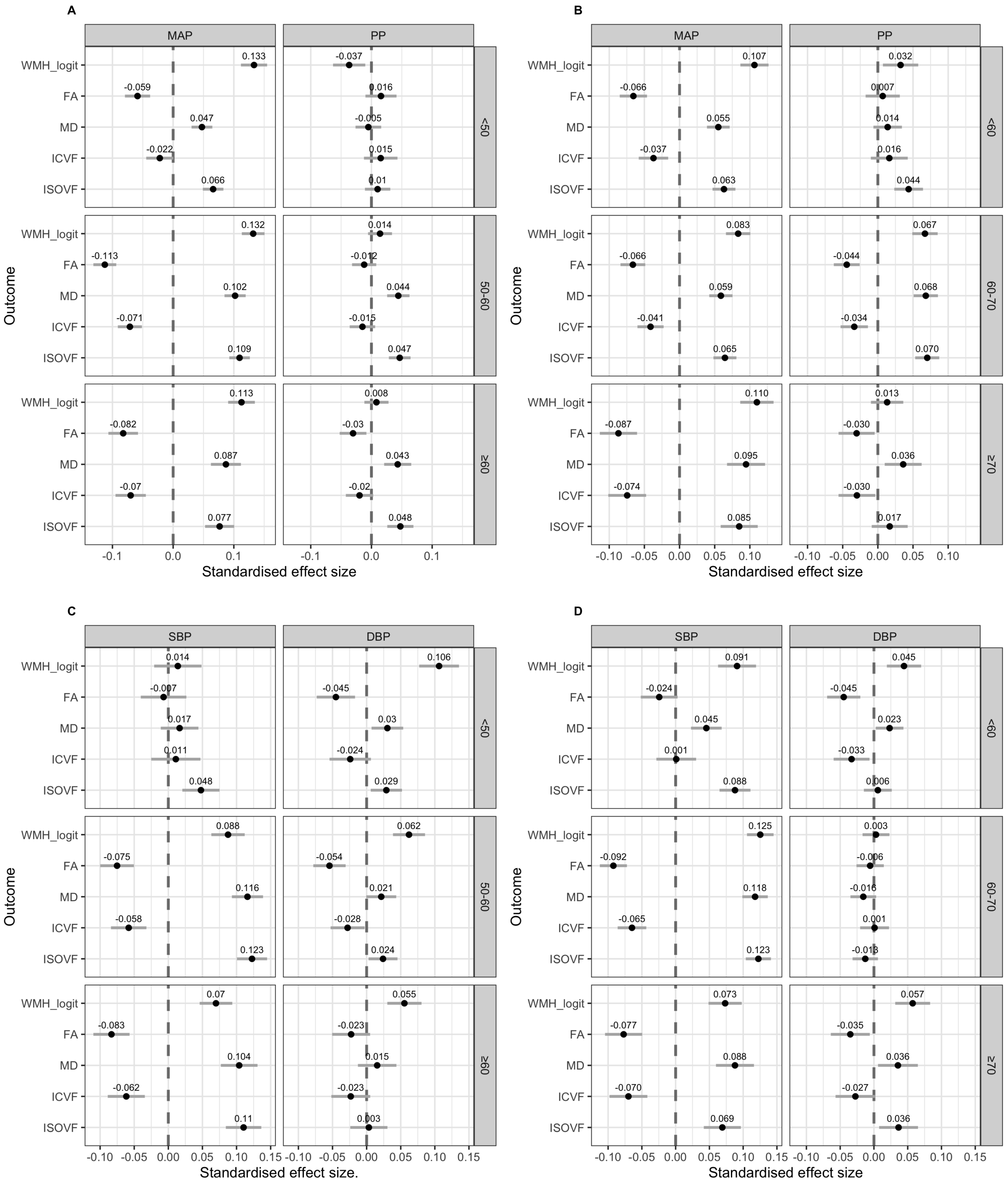

Figure S11: Standardised coefficients for past and concurrent blood pressure in multivariable analyses adjusted for the other blood pressure measure, sex, smoking status, diabetes, source of blood pressure measurement, and assessment centre and stratified by age decade. The longitudinal analyses also were adjusted for the time between the baseline and follow-up visit. **A:** longitudinal analysis with past MAP and

PP, **B:** cross-sectional analysis with concurrent MAP and PP, **C:** longitudinal analysis with past SBP and DBP, **D**: cross-sectional analysis with concurrent SBP and DBP. Abbreviations: WMH_logit - logit-transformed white matter hyperintensity load, FA - Fractional Anisotropy, MD - Mean Diffusivity, ICVF - Intracellular Volume Fraction, ISOVF - Isotropic Compartment Volume Fraction.
