# Supplementary figures and images for: Blood Pressure Determinants of Cerebral White Matter Hyperintensities and Microstructural Injury: UK Biobank Cohort Study
Source: Hypertension. 2021 Jun 1;78(2):532–9. doi: 10.1161/HYPERTENSIONAHA.121.17403 (PMC8260341; doi:10.1161/HYPERTENSIONAHA.121.17403)

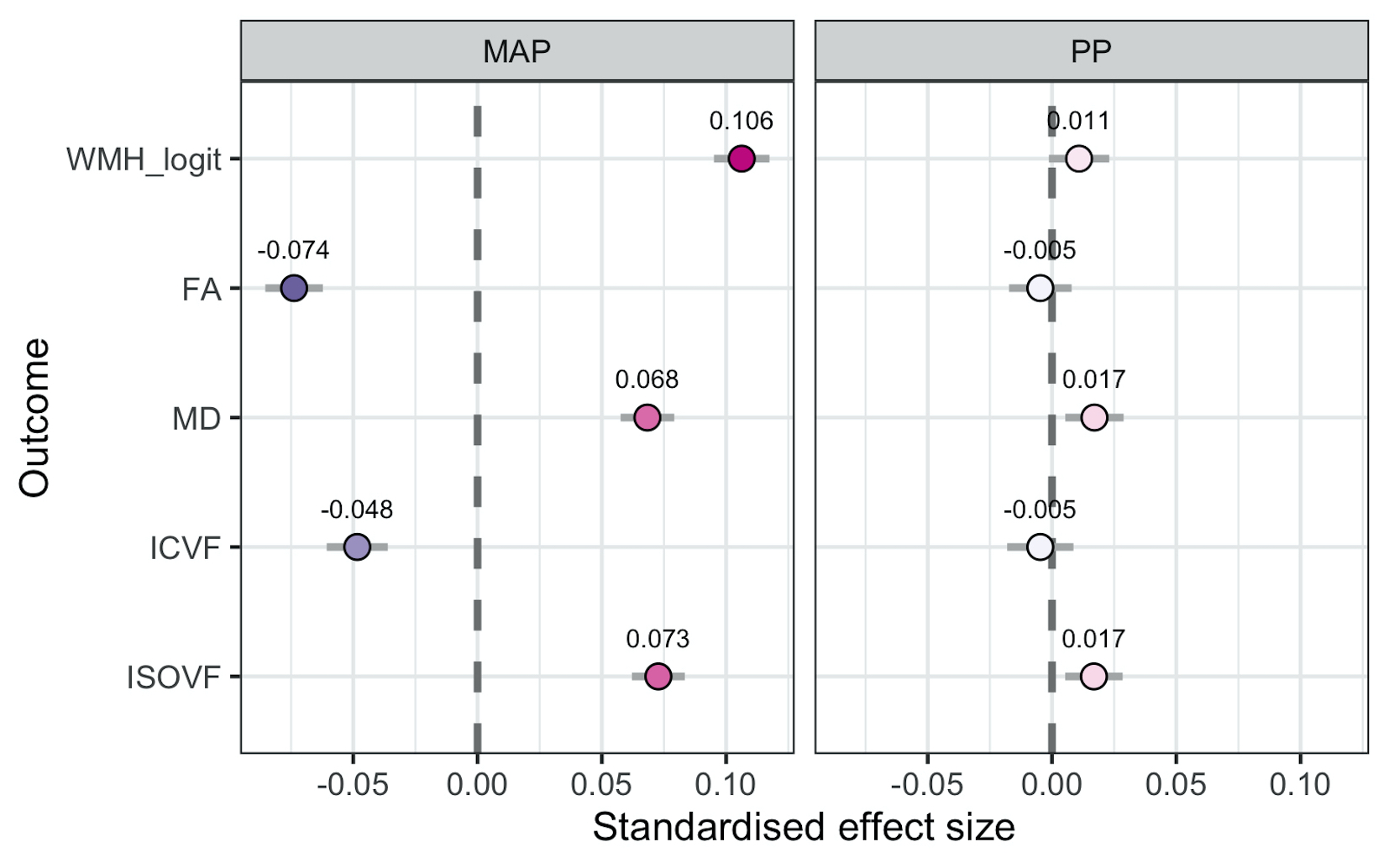

Supplement: Supplementary file 2 [file hyp-78-532-s002.jpg]
